# Solution-Processed Chloroaluminum Phthalocyanine (ClAlPc) Ammonia Gas Sensor with Vertical Organic Porous Diodes

**DOI:** 10.3390/s21175783

**Published:** 2021-08-27

**Authors:** Govindsamy Madhaiyan, An-Ting Sun, Hsiao-Wen Zan, Hsin-Fei Meng, Sheng-Fu Horng, Li-Yin Chen, Hsiao-Wen Hung

**Affiliations:** 1 Institute of Physics, National Yang Ming Chiao Tung University, Hsinchu 30010, Taiwan; meng@nctu.edu.tw; 2 Department of Electrical Engineering, National Tsing Hua University, Hsinchu 30010, Taiwan; anton0975667921@gmail.com (A.-T.S.); sfhorng@ee.nthu.edu.tw (S.-F.H.); 3 Department of Photonics, Institute of Electro-Optical Engineering, College of Electrical and Computer, Engineering, National Yang Ming Chiao Tung University, Hsinchu 30010, Taiwan; hsiaowen@mail.nctu.edu.tw; 4 Intelligent Energy-Saving Systems Division, Green Energy and Environment Research Laboratories, Industrial Technology Research Institute, Hsinchu 30010, Taiwan; htxwen@itri.org.tw

**Keywords:** organic semiconductor, vertical organic diode, e-nose, ammonia gas sensor, room temperature

## Abstract

In this research work, the gas sensing properties of halogenated chloroaluminum phthalocyanine (ClAlPc) thin films were studied at room temperature. We fabricated an air-stable ClAlPc gas sensor based on a vertical organic diode (VOD) with a porous top electrode by the solution process method. The surface morphology of the solution-processed ClAlPc thin film was examined by field emission scanning electron microscopy (FESEM) and atomic force microscopy (AFM). The proposed ClAlPc-based VOD sensor can detect ammonia (NH_3_) gas at the ppb level (100~1000 ppb) at room temperature. Additionally, the ClAlPc sensor was highly selective towards NH_3_ gas compared to other interfering gases (NO_2_, ACE, NO, H_2_S, and CO). In addition, the device lifetime was tested by storing the device at ambient conditions. The effect of relative humidity (RH) on the ClAlPc NH_3_ gas sensor was also explored. The aim of this study is to extend these findings on halogenated phthalocyanine-based materials to practical electronic nose applications in the future.

## 1. Introduction

In recent years, the electronic nose (e-nose) has received considerable attention due to its potential applications in several fields including environmental monitoring, food storage, healthcare, industry, automobiles, military, and cosmetics [1,2,3,4,5]. An e-nose is an analytical tool that can sense and identify odors and flavor using a sensor array. For an e-nose system, the choice of reliable gas sensors is the key factor for integration. Based on their working principle, different types of gas sensors have been demonstrated for their related applications [6,7]. Optical, acoustic, electrochemical, and catalytic sensors have shown some limitations such as a high cost, huge size, heavy weight, low reproducibility, poor selectivity, and complex system. In this context, solid-state gas sensors are appropriate candidates for developing a compact e-nose technology due to their low production cost, easy handling, good sensing behavior, and device miniaturization [5].

Recently, Ali et al. [1] reviewed e-nose technology for the quality testing of food and agricultural products. It can be seen that metal oxide semiconductors (MOS) have been widely used in solid-state gas sensors to detect a wide variety of gases including flammable and toxic gases. Apart from the advantages of MOS sensor arrays, there are still some challenges for developing a portable e-nose with MOS sensor arrays. For instance, most MOS sensor arrays require a high operating temperature, which consumes high energy and takes extra time for heating before device operation [3]. To realize a reliable e-nose system, a lot of aspects including good sensitivity/selectivity, low power consumption, room temperature operation, high stability, compactness, and cost effectiveness should be considered [5]. Over recent decades, several groups have investigated organic semiconductor (OSC)-based gas sensor arrays for e-nose applications [8,9,10,11,12,13]. OSC-based gas sensors are a benefit to e-nose systems owing to their inherent properties such as room temperature operation, fast response/recovery, low manufacturing cost, light weight, low power consumption, wide variety of material choice, and mechanical flexibility. In our prior works, we also successfully demonstrated numerous air-stable OSC gas sensors with room temperature operation for healthcare [14,15,16], environmental [17,18,19], and food quality monitoring [20]. In addition, we proposed an ultra-low power gas sensor based on a vertical organic diode (VOD) to detect ppb-level ammonia (NH_3_) at room temperature [21,22]. The detection of NH_3_ is very crucial because of its increasing utilization in multiple fields such as health and environmental monitoring, food quality testing, agricultural production, and industrial applications. For example, in healthcare monitoring, NH_3_ in exhaled human breath has been considered as a biomarker for chronic kidney diseases (CKD). Other volatile amine gases such as trimethylamine and dimethylamine have been demonstrated as important indicators for fish freshness [20]. In addition, only limited OSC gas sensor arrays currently fulfill the requirements of e-nose applications. Therefore, more explorations on environmentally stable OSC materials are still needed.

Phthalocyanines (Pcs) are one type of robust semiconducting, organic, small molecule material which can be considered a candidate for e-nose applications. In prior reports, Pcs have been utilized in various applications including field effect transistors [23], nonlinear optics [24], chemical sensors [25,26,27,28], photovoltaics [29], and heterojunction devices [30] due to their simple synthesis, non-toxicity, and chemical/thermal stability. Additionally, a variety of MPcs (e.g., CuPc, PbPc, ZnPc, FePc, CoPc) have been used as active layers in gas sensors, which show good sensitivity to both electron acceptor and donor gases [31,32,33,34,35]. It was noted that the MPc active layers were mostly prepared by vacuum or thermal evaporation because of their insolubility in conventional solvents.

In the present work, chloroaluminum phthalocyanine (ClAlPc) was used as the sensing layer to detect ppb-regime NH_3_ gas at room temperature. According to the literature, halogenated Pcs exhibit a favorable morphological structure and high thermal stability compared to metal Pcs [36,37]. In a few studies, ClAlPc and its composites have been utilized in gas and humidity sensors [38,39,40,41,42,43,44,45,46]. In 2011, Azim-Araghi et al. [42] studied the effect of temperature and humidity on a ClAlPc-based O_2_ sensor. Similarly, Jafari et al. [41] found the sensing response of a ClAlPc sensor (NH_3_ and ethanol) to be enhanced with the increase in the temperature up to the optimal operating temperature (T_max_ = 76 °C), and the sensing response gradually reduced at high temperatures (>85 °C) due to the adsorption/desorption equilibrium phenomenon [43]. Recently, Araghi et al. [46] fabricated a CO_2_ gas sensor using ClAlPc by thermal evaporation. However, all the reported ClAlPc gas sensors were demonstrated to realize as low as ppm-regime sensitivity. In this paper, we explored the basic sensing properties of a ClAlPc-based VOD sensor, which exhibited a ppb-regime NH_3_ sensitivity at room temperature. The proposed ClAlPc-based VOD sensor could be a potential candidate for a reliable e-nose system in the future. 

## 2. Experimental Details

### 2.1. Materials and Device Fabrication

**Figure 1:**
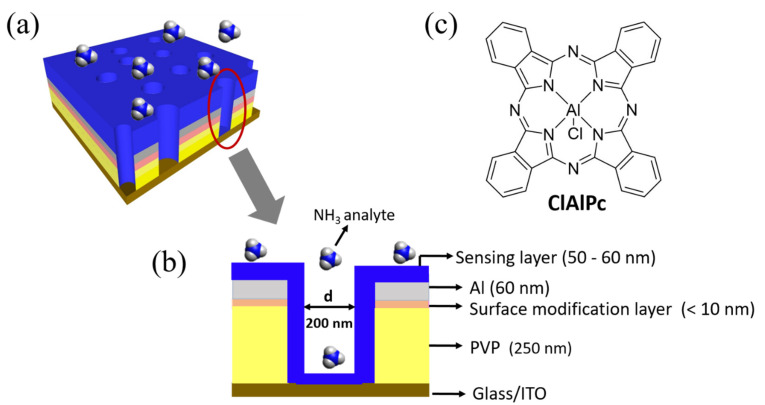
(**a**) Schematic illustration of fabricated vertical organic diode (VOD) sensor. (**b**) Cross-section view of the VOD device. (**c**) Molecular structure of ClAlPc.

The fabrication process flow of the proposed VOD sensor is shown in Figure S1, see Supplementary Materials. The proposed porous VOD sensor was fabricated on a pre-cleaned and oxygen plasma-treated (100 W, 600 s) indium tin oxide (ITO) glass substrate. Insulating cross-linkable poly (4-vinylphenol) (PVP) solution was spun (3500 rpm, 40 s) on the ITO substrate and annealed at 200 °C for 1 h (thickness: ~250 nm). Poly(3-hexylthiophene) (P3HT) solution (1.5 wt% in chlorobenzene) was spin coated (3500 rpm, 40 s) onto the PVP layer and annealed at 200 °C for 10 min as a surface modification layer to adsorb polystyrene (PS) nanospheres. PS nanospheres with a diameter of 200 nm were then coated for the following colloidal lithography process [18,19,20,21]. Aluminum 60 nm in thickness was thermally evaporated as the top electrode. The PS nanospheres were removed with 3M scotch adhesive tape to form a porous Al electrode. Afterwards, the region without the top Al electrode was etched by O_2_ plasma. Finally, the sensing material ClAlPc was dissolved in trifluoroacetic acid (0.1 wt%), spun (2000 rpm, 40 s) onto the porous structure, and annealed at 75 °C for 10 min (thickness: 50~60 nm) to fill into the vertical cylinders and to connect the top and bottom electrodes. The final structure of the fabricated VOD and its cross-section view are shown in Figure 1a,b. The molecular structure of ClAlPc is illustrated in Figure 1c. The active area of the ClAlPc VOD sensor was 1 mm^2^, defined by the cross-section of the patterned ITO and Al electrodes. 

### 2.2. Sensor Measurement System

In order to investigate the gas sensing and electrical properties of the proposed ClAlPc VOD sensor, we utilized our standard sensor measurement system. More details of our sensing system have been reported in our prior works [19,21]. The target gas was introduced into a sensing chamber through a gas-tight syringe to interact with the sensor device. The carrier gas (air) was well mixed with the target gas before reaching the sensing chamber. The gas flow was controlled by a pump (flow rate: 500 mL/min). For our measurement, we fixed the gas injection period at 60 s for each concentration. The gas concentration can be accurately controlled by adjusting the injection rate of the electrical syringe pump system. A constant relative humidity (RH = 10%) was maintained throughout the measurement with the help of a NaOH cylinder. For comparative study, the device was also tested at different humidity conditions (RH = 10%, 20%, 50%, 70%). In this case, the NaOH cylinder was removed, and the flow rate was controlled by a mass flow controller in the system. The current–voltage characteristics of the sensor were measured by a Keithley Source Meter (model 2400) and a Keysight U2722A USB Modular Source Measure Unit. The sensing response was calculated using the following formula: R = (ΔI/I_initial_), i.e., the current variation ratio within the sensing time divided by the initial current, where R is the sensing response, ΔI is the current difference at a fixed sensing time (60 s), and I_initial_ is the initial current.

## 3. Results and Discussions

### 3.1. Morphology Analysis

**Figure 2:**
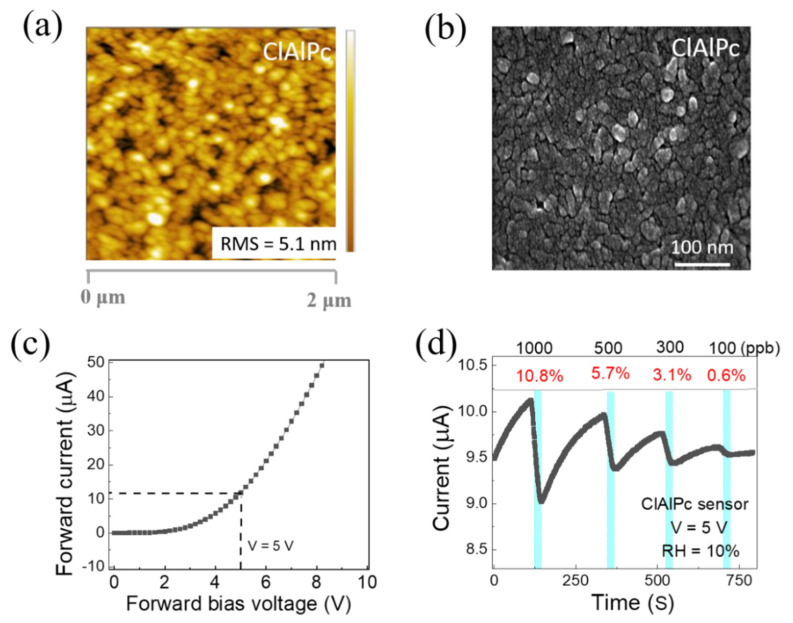
(**a**) AFM and (**b**) SEM images of ClAlPc film. (**c**) Current–voltage characteristic of ClAlPc sensor. (**d**) Representative real-time current of ClAlPc device exposed to ammonia concentrations of 1000, 500, 300, and 100 ppb, sequentially.

The surface morphology of the solution-processed ClAlPc thin film was investigated with the aid of a field emission scanning electron microscope (SEM SU8010) and an atomic force microscope (Bruker Edge). Figure 2a,b show the AFM and FESEM images of the ClAlPc thin film deposited on a glass substrate via spin coating (same method used for the device fabrication). The root mean square (RMS) value of the surface roughness was 5.1 nm, indicating a uniform surface morphology. As shown in Figure 2b, the grains are uniformly distributed, and the average grain size is between 42 and 50 nm.

### 3.2. Electrical and Sensing Behavior

The current–voltage characteristic of the ClAlPc sensor is depicted in Figure 2c. The ClAlPc VOD sensor showed good diode behavior. The real-time measurement of the ClAlPc sensor was conducted with a fixed operating voltage of 5 V under various concentrations of NH_3_ (100–1000 ppb) at room temperature. Figure 2d displays the representative current in real-time measurements. The cyan color periods in Figure 2d indicate the time under the exposure of NH_3_ (60 s). In general, when OSC-based chemical sensors are exposed to analytes, specific interactions (e.g., doping/de-doping, or trapping/quenching of charge carriers) occur between the sensing layer and the analyte. These interactions lead to affecting the charge transport properties (charge carrier density or mobility) in the OSC sensing layers [14,15,20,46,47,48,49]. As shown in Figure 2d, the current of the ClAlPc device obviously decreases when the sensor is exposed to NH_3_ gas and increases after the removal of NH_3_ gas from the sensing chamber. According to prior reports, the reducing NH_3_, due to its lone pair of electrons, acts as a de-doping agent for p-type semiconductors. Thus, for ClAlPc, which is a p-type OSC [47], the hole carrier density may be decreased under the exposure of NH_3_ [47,48,49,50]. As a result, the current (in the cyan color periods) decreases upon different concentrations of NH_3_ exposure (100~1000 ppb) and recovers when removing NH_3_ from the chamber. The sensing responses to 1000, 500, 300, and 100 ppb NH_3_ are 10.8%, 5.7%, 3.1%, and 0.6%, respectively. To know the effect of humidity on the ClAlPc NH_3_ gas sensor, we tested the device under different relative humidity conditions (RH = 10~70%). The sensing response gradually increases while varying the humidity of the sensing system, as shown in Figure S2. For instance, at RH = 10%, the responses to 1000, 500, 300, and 100 ppb NH_3_ were 10.8%, 5.7%, 3.1%, and 0.6%, respectively. At RH = 70%, the responses to 1000, 500, 300, and 100 ppb NH_3_ were 20.4%, 15.5%, 10.6%, and 5.9%, respectively. Similar results have also been seen in other organic semiconductor materials such as DPA-Ph-DBPzDCN and polyaniline [51,52]. However, more studies are needed to further understand the mechanism between relative humidity and NH_3_ sensing in ClAlPc in the future. It is worthy to note that the response curves exhibit good linearity under different humidity conditions, indicating the sensor can provide a convincing sensing capability, if a calibration is conducted under the test circumstance in advance, or the measuring system is equipped with some humidity control part (e.g., NaOH cylinder) to maintain a constant humidity.

### 3.3. Selectivity and Repeatability

**Figure 3:**
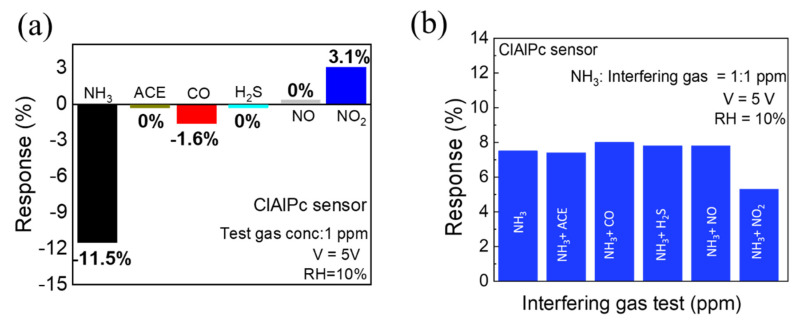
(**a**) Selectivity test (test gas concentration = 1 ppm) of ClAlPc sensor. (**b**) NH_3_ sensing and its cross-sensitivity towards different gases (ACE, CO, H_2_S, NO, and NO_2_) with equal concentrations (interfering gases: NH_3_ = 1:1 ppm).

In gas sensor technology, selectivity and repeatability are two important points to evaluate in sensors for real-time applications. To study the selectivity of the ClAlPc sensor, the sensor was exposed to various gases separately at the same concentration (1 ppm). As shown in Figure 3a, the device shows a high response when exposed to NH_3_ (11.5%), whereas the response to NO_2_ and CO is only 3.1% and 1.6%, respectively. Interestingly, the ClAlPc sensor shows no response to ACE, H_2_S, and NO gases. Furthermore, we also exposed the ClAlPc device to NH_3_ and other interfering gases (ACE, CO, H_2_S, NO, and NO_2_) at the same ratio of concentrations (1:1 ppm) simultaneously. As shown in Figure 3b, there is no significant change in NH_3_ sensing when mixed with other interfering gases (CO, H_2_S. NO, ACE), except for NO_2._ Therefore_,_ the ClAlPc sensor can be employed for the detection of NH_3_ gas even in harsh environments. As shown in Figure S4, even the ClAlPc sensor stored for 30 days exhibits a stable response in repeated test cycles. These results suggest that the proposed ClAlPc sensor exhibits good selectivity as well as repeatability.

### 3.4. Long-Term Stability

**Figure 4:**
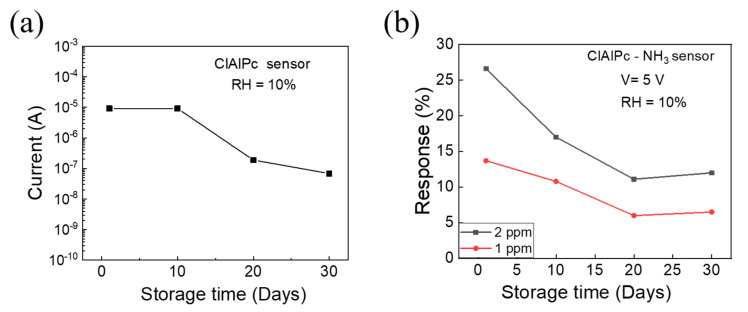
(**a**) The device current at an operating voltage of 5 V, and (**b**) NH_3_ sensing response of ClAlPc sensor on different days. The ClAlPc sensor was stored at ambient conditions.

Finally, the long-term stability of the ClAlPc NH_3_ sensor was determined, where the senor was stored in ambient air. The device current at an operating voltage of 5 V and the NH3 sensing response of the ClAlPc sensor were traced as a function of the storage days, which are shown Figure 4a,b, respectively. After one month, the current in the ClAlPc device decreased from 9.2 × 10^−6^ to 6.8 × 10^−8^ A. Initially, the sensing response decreased and then maintained a constant level after 20 days, as shown in Figure 4b. To understand the degradation mechanism of the gas sensor, further analysis will be carried out in the future. However, the performance of the proposed solution-processed ClAlPc sensor showed a better performance compared with previously reported ClAlPc film sensors (Table 1). In particular, our proposed ClAlPc VOD sensor exhibits ppb-regime NH_3_ sensitivity at room temperature. The nanoporous diode with a vertical structure demonstrates effective gas sensing because the nanopores act as direct pathways for gas molecules to interact deeply with the ClAlPc sensing layer. Therefore, the sensor provides a good selectivity, good repeatability, and reasonable sensing response even after 30 days.

## 4. Conclusions

In this study, we fabricated a solution-processed vertical organic diode NH_3_ sensor using ClAlPc as the sensing layer. The proposed sensor was operated at room temperature to detect NH_3_ gas at a ppb regime with a fixed operating voltage of 5 V. The solution-processed ClAlPc thin film exhibited a good surface morphology. The ClAlPc sensor showed good selectivity to NH_3_ gas, and the sensing capability could be sustained over a month at ambient conditions. The device also showed a stable performance in the test with repeated cycles. Moreover, the effect of the relative humidity (RH = 10~70%) on the ClAlPc sensor was studied. The ClAlPc ammonia sensor satisfies all the basic requirements for a reliable gas sensor owing to its good selectivity, sensitivity, repeatability, stability, and room temperature operation. These results suggest that a porous vertical organic diode utilizing ClAlPc as the sensing film will be a potential candidate for electronic nose applications.

## Figures and Tables

**Table 1:** Comparison of reported gas sensors based on chloroaluminum phthalocyanine.

Material	Film Formation	Target Gas	Operating Temperature (°C)	Lifetime	Reference
ClAlPc	Thermal evaporation	NO_2_, Cl_2_, NH_3_ (500 ppm)	RT~106 °C	NA	40
ClAlPc	Thermal evaporation	NH_3_, Ethanol (100 ppm)	RT~175 °C	>60 days	41
PAni-ClAlPc	Spin coating	NO_2_ (10 ppm)	RT~76.8 °C	NA	42
PdPc/ClAlPc	Thermal evaporation	O_2_ (%)	RT~76.8 °C	~60 days	43
PAni-ClAlPc	Spin coating	CO_2_ (500 ppm)	RT	NA	46
ClAlPc	Thermal evaporation	CO_2_ (1000 ppm)	RT~176 °C	NA	47
**ClAlPc**	**Spin coating**	**NH_3_ (100 ppb)**	**RT**	**>30 days**	**This work**

## Supplementary Materials

The following are available online at https://www.mdpi.com/article/10.3390/s21175783/s1.
