# Supplementary material for: Solution-Processed Chloroaluminum Phthalocyanine (ClAlPc) Ammonia Gas Sensor with Vertical Organic Porous Diodes
Source: Sensors (Basel). 2021 Aug 27;21(17):5783. doi: 10.3390/s21175783 (PMC8433672; doi:10.3390/s21175783)
Supplement: Supplementary file 1 [file sensors-21-05783-s001.zip › Updated Sup.pdf]

Supporting information

Solution-processed Chloroaluminum Phthalocyanine (ClAlPc) Ammonia Gas Sensor with Vertical Organic Porous Diodes

Govindsamy Madhaiyan [1, *], An-Ting Sun[3], Hsiao-Wen Zan[2], Hsin-Fei Meng[1], Sheng-Fu Horng[3], Li-Yin Chen[2, *], Hsiao-Wen Hung[4]

[1] Institute of Physics, National Yang Ming Chiao Tung University, Hsinchu, Taiwan.

[2] Department of Photonics, Institute of Electro-Optical Engineering, College of Electrical and Computer Engineering, National Yang Ming Chiao Tung University, Hsinchu, Taiwan.

[3] Department of Electrical Engineering, National Tsing Hua University, Taiwan

[4] Intelligent Energy-Saving Systems Division, Green Energy and Environment Research Laboratories, Industrial Technology Research Institute, Hsinchu, Taiwan,

Corresponding authors

govindaswamy01@gmail.com (G. Madhaiyan), lychen@nycu.edu.tw (L.-Y. Chen)

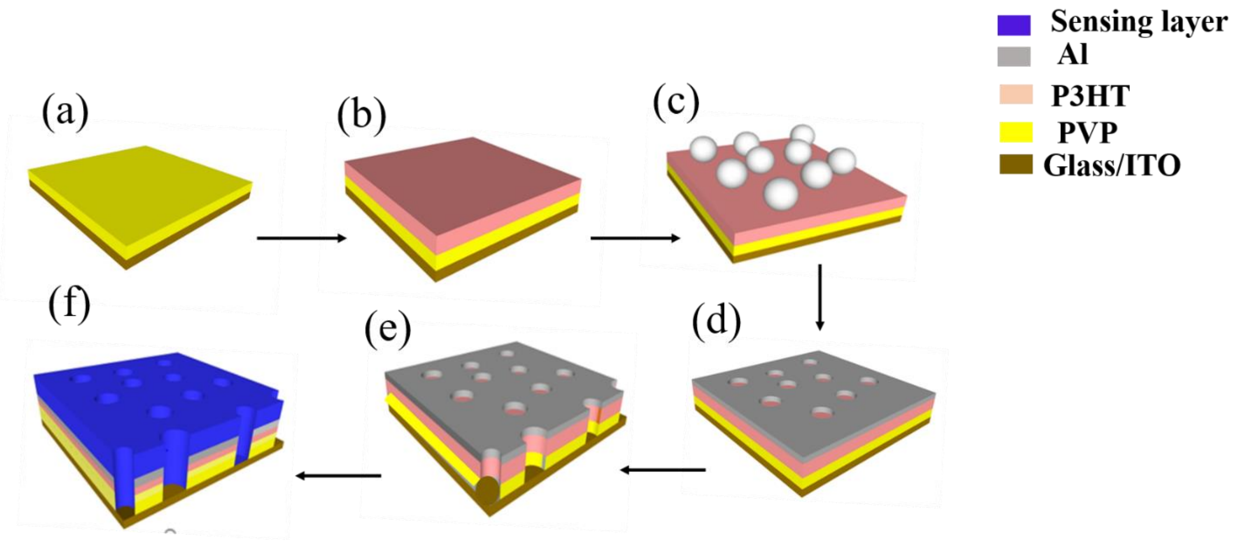

Figure S1 The fabrication process flow of the proposed VOD sensor. (a) PVP and (b) P3HT surface modification layer was formed on ITO glass substrate by spin coating. (c) PS (Polystyrene) nanosphere formation. (d) Top electrode deposition by thermal evaporation; the PS spheres were removed by scotch tape. (e) O_2 plasma etching. (f) ClAlPc sensing material was spin coated onto the substrate to complete the sensor fabrication.

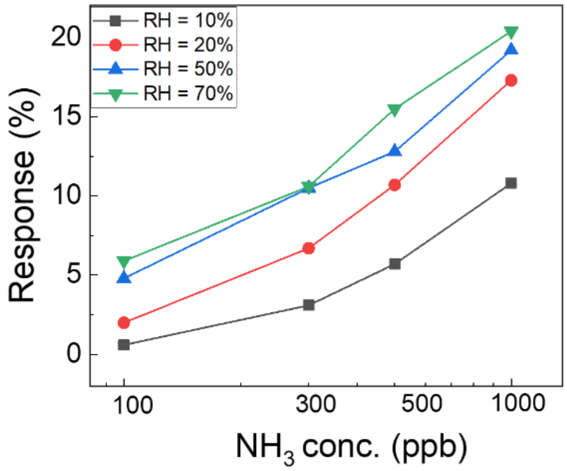

Figure S2 Sensing response as a function of NH_3 concentration at different RH level (10% ~ 70%)

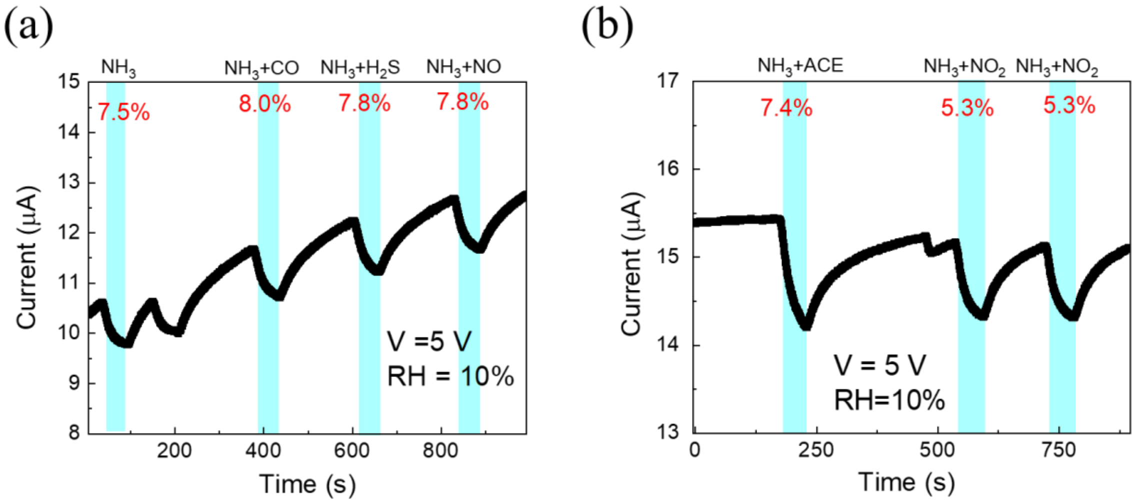

Figure S3 The real-time NH_3 sensing measurement of ClAlPc device with different interfering gases (Interfering gases: NH_3 = 1:1 ppm). (a) pure NH_3, NH_3+CO, NH_3+H_2S, and NH_3+NO. (b) NH_3+ACE, NH_3+NO_2, and NH_3+NO_2.

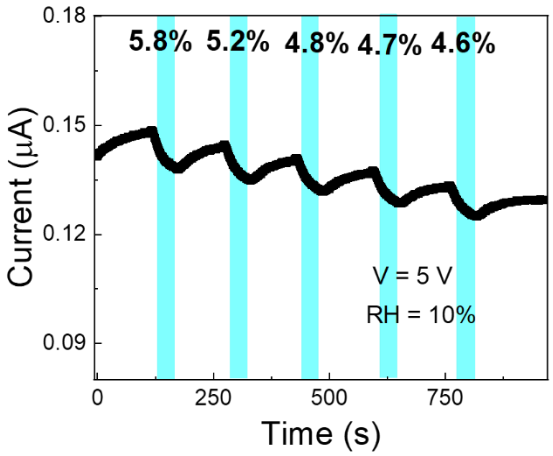

Figure S4 Repeatability of ClAlPc sensor (after one month) for 5 cycles with fixed concentration of 1 ppm NH_3 gas.
